# ABO-Incompatible Robotic-Assisted Kidney Transplantation in the Obese Recipient

**DOI:** 10.3389/fsurg.2020.00049

**Published:** 2020-08-07

**Authors:** Thomas Prudhomme, Arnaud Del Bello, Federico Sallusto, Marine Lesourd, Nassim Kamar, Nicolas Doumerc

**Affiliations:** ^1^Department of Urology, Kidney Transplantation and Andrology, Toulouse Rangueil University Hospital, Toulouse, France; ^2^Department of Nephrology and Organ Transplantation, Toulouse Rangueil University Hospital, Toulouse, France

**Keywords:** robotic-assisted kidney transplantation, ABO-incompatible, open kidney transplantation, desensitization protocols, delayed graft function

## Abstract

**Objective:** The objective of this preliminary study was to report and compare the peri-operative and functional results of ABO-incompatible (ABOi) living-donor robotic-assisted kidney transplantation (RAKT), ABO-compatible (ABOc) living-donor RAKT, and ABOi living-donor open kidney transplantation (OKT).

**Materials and Methods:** For the present retrospective study, we analyzed data of consecutive patients who underwent ABOi or ABOc-RAKT and ABOi-OKT, from January 2015 to December 2019, in one French academic center. Patients' baseline characteristics, operative, and functional outcomes were compared between ABOi-RAKT, ABOc-RAKT, and ABOi-OKT.

**Results:** 29 RAKT, including 7 ABOi-RAKT, and 56 ABOi-OKT were performed in our center. Median follow-up was 2.0 years. Median recipient age, pre-emptive kidney transplantation rate, sex ratio and desensitization procedures were similar in ABOi-RAKT, ABOc-RAKT, and ABOi-OKT groups. Recipient BMI at transplantation was statistically higher in ABOi and ABOc-RAKT groups compared to ABOi-OKT. The surgical site complication (principally infection-related) rate was lower in ABOi-RAKT, without statistical differences (0 vs. 8.9%, respectively, in ABOi-RAKT and ABOi-OKT, *p* = 0.7). The delayed graft function rate was 0% in ABOi-RAKT, 13.6% in ABOc-RAKT, and 10.7% in ABOi-OKT (*p* = 0.6). The post-transplantation blood transfusion rate was statistically higher in the ABOi-OKT group (14.3 vs. 13.6 vs. 57.1% in ABOi-RAKT, ABOc-RAKT, and ABOi-OKT, respectively, *p* = 0.001). The kidney graft survival at 1 month and at last follow-up was not different between ABOi-RAKT and ABOi-OKT.

**Conclusion:** Our data support the use of ABOi-RAKT to restore accessibility to kidney transplantation for obese patients to the greatest extent possible. Large series are required to confirm these encouraging data from a single center.

## Introduction

Kidney transplantation (KT) is the best treatment for obese patients suffering from end-stage renal disease, but remains a daily surgical challenge (1). Several studies reported technical difficulties with traditional open approaches and a higher surgical post-operative complication rate, including wound dehiscence, surgical site infection, and lymphocele formation, in obese recipients (1, 2). Consequently, many transplant centers tend to contraindicate KT in obese recipients. However, compared to remaining on a waiting list, KT in obese recipients improves long-term survival (3) and enhances quality of life (4). Furthermore, in addition to obesity-related surgical difficulties, it is sometimes necessary to cross over immunological barriers in order to allow access to transplantation for obese patients. Then, ABO-incompatible (ABOi) living-donor kidney transplantation has been developed in order to reduce waiting times for deceased-donor kidney transplantation for transplantation candidates (5), in countries where a donor swap or a donor-chain program is not feasible. Despite excellent long-term reported outcomes (6), post-operative surgical complications, especially bleeding complications, were more frequent after ABOi living-donor kidney transplantation compared to ABO-compatible (ABOc) living-donor kidney transplantation (7).

In recent years, robotic-assisted kidney transplantation (RAKT) has been developed to reduce the surgical morbidity of KT. Thus, the feasibility, reproducibility, and safety of RAKT has been confirmed when performed by skilled robotic surgeons (8, 9).

The objective of this preliminary study was to report and compare the peri-operative and functional results of ABOi living-donor RAKT, ABOc living-donor RAKT, and ABOi living-donor open KT (OKT).

## Materials and Methods

### Patients and Database

For the present retrospective study, we analyzed data of consecutive patients who underwent ABOi or ABOc living-donor RAKT and ABOi living-donor OKT, from January 1, 2015 to December 31, 2019, in one French academic center. All living-donor RAKT were performed by a surgeon experienced in robotic surgery and ABOi living-donor OKT were performed by a surgeon experienced in OKT. The follow-up was performed in our center.

### Surgical Procedures

All living donor-nephrectomies (LDN) were minimally invasive surgical procedures (robotic-assisted LDN or pure laparoscopic LDN).

A RAKT was performed in obese patients contraindicated for OKT. All ABOi and ABOc living-donor RAKT were performed by an experienced robotic surgeon (ND) and all ABOi-OKT were performed by an experienced KT surgeon (FS).

**Figure 1:**
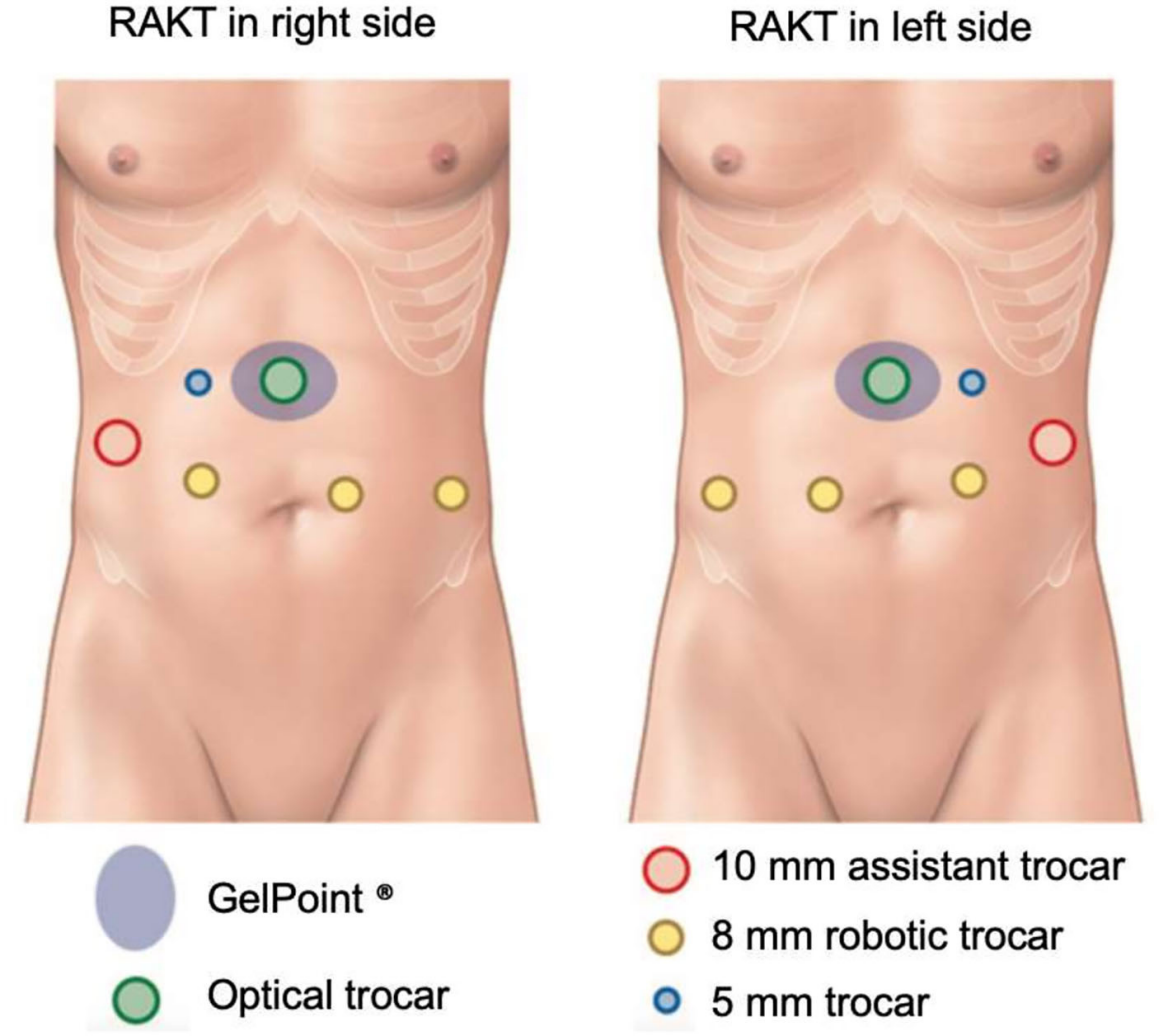
RAKT trocar location. Adapted from Prudhomme et al. (12).

The surgical procedure of RAKT has been previously described (10–12). Briefly, we performed RAKT with a 4-arm Si HD Da Vinci^®^ (13). Usually, a 7-cm upper-midline incision was performed to introduce the graft through an Alexis® retractor. A 12-mm optical trocar was inserted through the Alexis® retractor and two 8-mm trocar were inserted along the left and right para-median lines (Figure 1). After dissection of the external iliac vessels, a peritoneal flap was created for the final retroperitonealization of the kidney, to simplify the performance of post-operative biopsies. End-to-side venous and arterial anastomoses were performed with Gore-Tex® PTFE 5/0 and 6/0, respectively. The pneumoperitoneum pressure during vascular anastomosis was 12 mmHg. After reperfusion, pressures were reduced to 7 mmHg. The donor ureter was anastomosed to the bladder mucosa, using the Lich-Gregoir technique.

In ABOi-RAKT, because bleeding is a major complication due to desensitization protocols, we did not use intra-operative heparinization during vascular anastomoses. An ABOi-KT was performed in the absence of an ABO-compatible donor.

### Desensitization Protocols and Maintenance Immunosuppression

Desensitization protocols included plasmapheresis or specific immunoadsorption. The induction therapy included Rituximab (375 mg/m^2^, one injection), polyclonal antibodies (lymphoglobulins, Grafalon®, 9 mg/kg, one injection) or anti-IL2R blockers (basiliximab, Simulect®, 20 mg at day 0 and 4), according to the period of transplantation, for ABOi-RAKT or ABOi-OKT and anti-IL2R blockers or no induction (according to presence of preformed anti-HLA non-donor-specific antibodies at transplantation) for ABOc-RAKT. The maintenance immunosuppression included tacrolimus and steroids, associated with everolimus or mycophenolic acid.

### Study Variables and Outcomes

In the database of consecutive patients, the patients' baseline characteristics that were collected included: recipient age (years), recipient sex, recipient body mass index (BMI) (kg/m^2^) at transplantation, pre-emptive KT rate, desensitization procedures and induction therapy. The intra-, post-operative, and functional outcomes were: cold ischemia time (minutes), operative time (minutes), rewarming time (minutes), delayed graft function rate, surgical site infection rate, post-transplantation blood transfusion rate, length of hospital stay (days), 1-year post-transplantation rejection rate, 1-year patient survival rate, 1-month kidney graft survival rate, last-follow-up kidney graft survival rate, and median follow-up.

Cold ischemia time was defined as the duration of cold storage, with or without perfusion, with a storage solution, before graft introduction into the recipient. Rewarming time was defined as the duration between insertion of kidney graft into the abdominal cavity and graft reperfusion.

### Statistical Analysis

Quantitative data were expressed as median (interquartile range) and qualitative data as number and proportion (%). Patients' baseline characteristics, intra-operative, post-operative, and functional outcomes were compared between ABOi-RAKT, ABOc-RAKT, and ABOi-OKT.

Quantitative values were compared using ANOVA tests. Qualitative values were compared with the Chi 2 test or Fisher's exact test. The kidney graft survival was estimated using the Kaplan-Meier method and compared with the log-rank test method. All reported *p*-values were two-sided with a significance level at *p* < 0.05. A statistical analysis was performed using S Prism 7.0a (GraphPad Software Inc., La Jolla, CA, USA).

## Results

### Patients' Characteristics and Desensitization Protocols

From January 1, 2015 to December 31, 2019, we performed 29 living-donor RAKT, including 7 ABOi living-donor RAKT, and 56 ABOi living-donor OKT in our center. The patients' characteristics are shown in Table 1. Median recipient age, pre-emptive KT rate and sex ratio were similar in ABOi-RAKT, ABOc-RAKT, and ABOi-OKT groups. As expected, recipient BMI at transplantation was statistically higher in ABOi and ABOc-RAKT groups, compared to ABOi-OKT [33.1 (29.4–35.6) vs. 32.2 (30.1–35.0) vs. 23.0 (20.6–25.3) kg/m^2^ in ABOi-RAKT, ABOc-RAKT, and ABOi-OKT, respectively, *p* < 0.0001]. Desensitization procedures were similar in the ABOi-RAKT, ABOc-RAKT, and ABOi-OKT groups. Induction therapy rates were similar in ABOi-RAKT and ABOi-OKT, but induction therapy rate was statistically lower in the ABOc-RAKT group.

**Table 1:** Patients' characteristics and desensitization protocols.

		**ABOi-RAKT** **(*n* = 7)**	**ABOc-RAKT** **(*n*= 22)**	**ABOi-OKT** **(*n* = 56)**	***p***
**Recipient age (year)**					
	Median (IQR)	58.7 (45.8–70.5)	57.4 (42.6–62.9)	49.8 (38.1–59.5)	0.1
**Pre-emptive KT**					
	Yes, *n* (%)	5 (71.4%)	10 (45.5%)	25 (44.6%)	0.4
**Recipient sex**					
	Male (%)	6 (85.7%)	15 (68.2%)	34 (60.7%)	0.4
**Recipient Body Mass Index at Tx (kg/m**^**2**^**)**					
	Median (IQR)	33.1 (29.4–35.6)	32.2 (30.1–35.0)	23.0 (20.6–25.3)	**<0.0001**
**Desensitization procedure**					
Plasmapheresis	Yes, *n* (%)	6 (85.7%)	/	37 (66.1%)	0.4
**Number of sessions**					
	Median (IQR)	3.5 (2.5–5.3)	/	5 (3–6)	0.5
Specific Immunoadsorption	Yes, *n* (%)	1 (14.3%)	/	22 (39.3%)	0.4
**Number of sessions**					
	Median (IQR)	1 (1–1)	/	1 (1–2)	/
**Induction therapy**					
Rituximab	Yes, *n* (%)	6 (85.7%)	2 (9.1%)	56 (100%)	**<0.0001**
Polyclonal antibodies	Yes, *n* (%)	6 (85.7%)	3 (13.6%)	44 (78.6%)	**<0.0001**
Basiliximab	Yes, *n* (%)	1 (14.3%)	6 (27.3%)	12 (21.4%)	0.7

*The bold values indicates p < 0.005*.

### Intra-, Post-operative Outcomes, and Functional Outcomes

**Figure 2:**
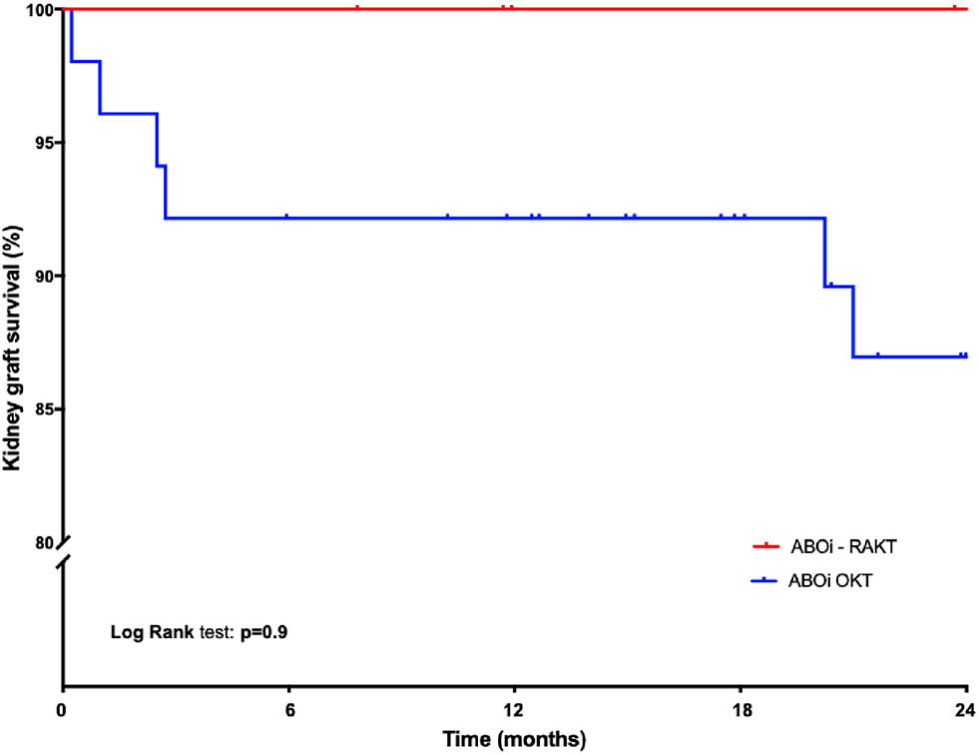
Kaplan-Meier kidney graft survival in the ABOi-RAKT and ABOi-OKT groups.

Cold ischemia time, operative time and rewarming time were statistically longer in the ABOi-OKT group (*p* < 0.0001) (Table 2). The surgical site complication (principally infection-related) rate was lower in ABOi-RAKT, without statistical difference (0 vs. 8.9% in ABOi-RAKT and ABOi-OKT, respectively, *p* = 0.7). The delayed graft function rate was 0% in ABOi-RAKT, 13.6% in ABOc-RAKT, and 10.7% in ABOi-OKT (*p* = 0.6) (Table 2). The post-transplantation blood transfusion rate was statistically higher in the ABOi-OKT group (14.3 vs. 13.6 vs. 57.1% in ABOi-RAKT, ABOc-RAKT, and ABOi-OKT, respectively, *p* = 0.001) (Table 2). Median length of hospital stay was 8 (7–10) days in ABOi-RAKT recipients, 9 (7–13) days in ABOc-RAKT and 8 (7–9) days in ABOi-OKT (*p* = 0.3). At 1 year of follow-up, the rejection rate, and patient survival rate were similar in the ABOi-RAKT, ABOc-RAKT, and ABOi-OKT groups. The kidney graft survival at 1 month and at last follow-up was not different between ABOi-RAKT and ABOi-OKT (Table 1 and Figure 2).

**Table 2:** Intra-, post-operative, and functional outcomes.

		**ABOi-RAKT** **(*n* = 7)**	**ABOc-RAKT** **(*n* = 22)**	**ABOi-OKT** **(*n* = 56)**	***p***
**Cold ischemia time (minutes)**					
	Median (IQR)	150.0 (120.0–150.0)	150.0 (140.0–155.0)	224.0 (205.0–267.0)	**<0.0001**
**Operative time (minutes)**					
	Median (IQR)	130.0 (110.0–190.0)	150 (126.0–165.0)	180 (135.0–210.0)	**<0.0001**
**Rewarming time (minutes)**					
	Median (IQR)	30.0 (25.0–45.0)	40.0 (28.8–47.5)	60.0 (45.0–77.8)	**<0.0001**
**Delayed graft function**					
	Yes, *n* (%)	0 (0%)	3 (13.6%)	6 (10.7%)	0.6
**Surgical site infection**					
	Yes, *n* (%)	0 (0%)	2 (9.1%)	5 (8.9%)	0.7
**Post-transplantation blood transfusion**					
	Yes, *n* (%)	1 (14.3%)	3 (13.6%)	32 (57.1%)	**0.001**
**One-year post-transplant rejection rate**					
	Yes, *n* (%)	2 (28.6%)	4 (18.2%)	9 (16.1%)	0.7
**One-year patient survival**					
	Yes, *n* (%)	7 (100%)	22 (100%)	51 (91.1%)	0.3
**One-month kidney graft survival**					
	Yes, *n* (%)	7 (100%)	20 (90.9%)	53 (94.6%)	0.6
**Kidney graft survival at last follow-up**					
	Yes, *n* (%)	6 (85.7%)	21 (95.5%)	45 (80.4%)	0.2
**Time between Tx and last follow-up (years)**					
	Median (IQR)	2.0 (1.0–3.0)	2.0 (0.9–2.8)	2.1 (1.3–3.3)	0.6

*The bold values indicates p < 0.005*.

## Discussion

Kidney transplantation is the best therapeutic option in patients with ESRD. In fact, survival is significantly higher in recipients compared to age-matched patients who are maintained on dialysis and age-matched patients who are awaiting KT (14). However, grade III obesity reduces the opportunity for male patients with ESRD to access transplantation (15).

Furthermore, it is sometimes necessary to overcome immunological barriers with desensitization strategies to allow access to transplantation for transplantation candidates. Thus, in countries where a donor swap or a donor-chain program is not feasible, ABO-incompatible programs have been developed (16).

Montgomery et al. (17) reported in 2012 the Data from the Scientific Registry of Transplant Recipients on ABOi living-donor kidney transplantation. They reported similar long-term patient survival between ABOi and ABOc matched recipients. However, they also reported a higher surgical post-operative complication rate and higher graft loss rate within 14 days of transplantation in ABOi recipients. Moreover, Naciri Bennani et al. (7) confirmed the higher risk of post-operative complications, especially bleeding complications in ABOi-KT.

Several teams reported their results on RAKT in obese recipients. The ERUS-RAKT group was created in 2015 in order to standardize the surgical procedure and prospectively evaluate data in the European centers involved in this project. Thus, Breda et al. (10, 11, 18), Territo et al. (8) confirmed the feasibility, reproducibility, and safety of RAKT when performed by skilled robotic surgeons. They reported low wound infection occurrence, a frequent complication of obese patients with open approaches.

Recently, Tzvetanov et al. (9), from the University of Illinois at Chicago group, reported their 10 years' experience of RAKT in obese patients. A total of 239 RAKT were performed with median BMI of 41.4 kg/m^2^. They reported a wound complication rate of 3.8% and optimal graft survival rate of 98 and 93% at 1 and 3 years of follow-up, similar with graft survival from patients undergoing OKT over the same time period from the UNOS database. They concluded that RAKT could improve access to KT in obese patients due to the low surgical complication rate.

In our preliminary study, although our population was limited, we reported optimal post-operative and functional results in ABOi-RAKT, with no delayed graft function and surgical site infection and low post-transplantation blood transfusion rate (14.3%). Moreover, we reported similar kidney graft survival in ABOi-RAKT (85.7%) and ABOi-OKT (80.4%) at 2 years of follow-up, and these survivals were similar with graft survival reported by de Weerd et al. (19), in their systematic review of ABOi kidney transplantation outcomes (96% at 1 year of follow-up). Our survivals were similar with graft survival reported by Tzvetanov et al. (9) (98% at 1 year) and Territo et al. (8) (98% at 1 year). Moreover, our median operative time in the ABOi and ABOc-RAKT groups (130 and 150 min) were shorter than those reported by Tzvetanov et al. (9) (289 min) and Territo et al. (8) (300 min), as was our rewarming time [30 and 40 min in the ABOi and ABOc-RAKT groups vs. 45 min reported by (9) and 60 min reported by (8)].

Thus, the Da Vinci® (Intuitive Surgical Inc., Sunnyvale, CA, USA) robotic platform, thanks to the EndoWrist® technology and the 3D vision it provides, allows precise vascular anastomoses to be performed while improving the surgeon's ergonomics and the quality of vision of the operating field. Consequently, magnified 3D vision and the lack of intra-operative heparinization during vascular anastomoses allow the rate of post-operative bleeding complications to be controlled in ABOi-RAKT.

## Conclusion

Our data support the use of RAKT combined with desensitization strategies to overcome immunological barriers, to restore accessibility to kidney transplantation for obese patients to the greatest extent possible. Large series are required to confirm these encouraging data from a single center.

## Data Availability Statement

The raw data supporting the conclusions of this article will be made available by the authors, without undue reservation.

## Ethics Statement

The studies involving human participants were reviewed and approved by ERUS-RAKT. The patients/participants provided their written informed consent to participate in this study.

## Author Contributions

TP, AD, and ND participated in research design, data analysis, and wrote the paper. FS, ML, and NK participated in research design, data analysis. All authors contributed to the article and approved the submitted version.

## Conflict of Interest

The authors declare that the research was conducted in the absence of any commercial or financial relationships that could be construed as a potential conflict of interest.
